# Premature Mortality from Cardiovascular Disease in the Americas – Will the Goal of a Decline of “25% by 2025” be Met?

**DOI:** 10.1371/journal.pone.0141685

**Published:** 2015-10-29

**Authors:** Pedro Ordunez, Elisa Prieto-Lara, Vilma Pinheiro Gawryszewski, Anselm J. M. Hennis, Richard S. Cooper

**Affiliations:** 1 Pan American Health Organization, Department of Noncommunicable Diseases and Mental Health, 525 23rd Street, NW, Washington, D.C., 20037, United States of America; 2 Loyola University Chicago, Stritch School of Medicine, Department of Public Health Sciences, Maywood, IL, 60153, United States of America; Chinese Academy of Medical Sciences, CHINA

## Abstract

**Background:**

Cardiovascular diseases (CVD) are the underlying cause 1.6 million deaths per year in the Americas, accounting for 30% of total mortality and 38% of by non-communicable deaths diseases (NCDs). A 25% reduction in premature mortality due four main NCDs was targeted by the 2011 High-level Meeting of the General Assembly on the Prevention and Control of NCDs. While overall CVD mortality fell in the Americas during the past decade, trends in premature CVD mortality during the same period have not been described, particularly in the countries of Latin America and the Caribbean.

**Methods:**

This is a population-based trend-series study based on a total of 6,133,666 deaths to describe the trends and characteristics of premature mortality due to CVD and to estimates of the average annual percentage of change during the period 2000–2010 in the Americas.

**Findings:**

Premature mortality due to CVD in the Americas fell by 21% in the period 2000–2010 with a -2.5% average annual rate of change in the last 5 year—a statistically significant reduction of mortality—. Mortality from ischemic diseases, declined by 25% - 24% among men and 26% among women. Cerebrovascular diseases declined by 27% -26% among men and 28% among women. Guyana, Trinidad and Tobago, the Dominican Republic, Bahamas, and Brazil had CVD premature mortality rates over 200 per 100,000 population, while the average for the Region was 132.7. US and Canada will meet the 25% reduction target before 2025. Mexico, Costa Rica, Venezuela, Dominican Republic, Panama, Guyana, and El Salvador did not significantly reduce premature mortality among men and Guyana, the Dominican Republic, and Panama did not achieve the required annual reduction in women.

**Conclusions:**

Trends in premature mortality due to CVD observed in last decade in the Americas would indicate that if these trends continue, the Region as a whole and a majority of its countries will be able to reach the goal of a 25% relative reduction in premature mortality even before 2025.

## Introduction

Cardiovascular diseases (CVD) are the underlying cause 1.6 million deaths per year in the Americas, accounting for 30% of total mortality and 38% of by non-communicable deaths diseases (NCDs) [1]. Reduction in the four main NCDs—CVD, cancer, chronic respiratory diseases, and diabetes- was targeted as crucial social responsibility of governments by the 2011 High-level Meeting of the General Assembly on the Prevention and Control of Non-communicable Diseases [2]. Chronic diseases and their risk factors—tobacco, alcohol, unhealthy diet, and lack of physical activity—constitute a threat to economic and social development throughout the world, and especially in low- and middle-income countries [3].

In 2013 the World Health Assembly adopted the NCD Global Monitoring Framework to strengthen the commitments undertaken by the world’s leaders regarding NCDs and their key risk factors. Prominent among the nine targets that were agreed upon was a 25% reduction in premature mortality (i.e., death among persons 30 to 69 years of age) caused by the four main NCDs by the year 2025 [4]. Considering the relative importance of CVD’s, and the proven impact of preventive interventions [5], there is no doubt that CVD prevention and control activities must play a central role in meeting these targets.

In the Americas, although CVD continues to be the leading cause of death [6], in the past decade (2000–2010) a decline in mortality of 19% was observed (20% among women and 18% among men). The greatest average annual reductions occurred in Canada (4.8%) and the United States (3.9%). In general, low- and medium-income countries had an excess of cardiovascular mortality at 56.7 and 20.6%, respectively, as compared to high-income countries [7].

While overall CVD mortality fell in the Americas during the past decade [7], trends in premature CVD mortality during the same period have not been described, particularly in the countries of Latin America and the Caribbean. For example, PAHO estimates that of the 1.6 million deaths caused by CVD every year in the Region, around a half million occur before age 70 [1]. Given the demographic and epidemiological changes that have occurred [8], these deaths can be considered avoidable. Historically premature mortality from CVD has been a key indicator of calls attention because it is less often embedded in multiple other co-morbidities in this age group. Trends in this age group are considered a particularly reliable indicator of the capacity of countries to implement effective public policies to prevent and postpone such illnesses, allowing access to quality services capable of offering timely detection and treatment of those who prematurely present with a CVD in the most productive period of life cycle.

The purpose of this study is to describe the demographic patterns of premature mortality from CVD at the present time, to determine the trends and characteristics of premature mortality due to CVD during the period 2000–2010 in the Americas, and to identify disparities.

## Methods

### Source of data and case definition

We extracted mortality data from the PAHO/WHO Regional Mortality database (updated in July 2013) which comprises deaths registered in national vital registration systems and reported annually to PAHO by national authorities in each country [9]. This data set contains information on sex, age, date of occurrence, place of residence and underlying cause of death, coded using the ICD-10.The United Nations Population Division was the source of mid-year population used to calculate rates [10]. The WHO World Standard Population was used to calculate age-adjusted mortality rates by direct standardization [11].

Deaths due to CVD were defined as all deaths whose underlying cause was classified in the Chapter IX, Diseases of Circulatory System (I00-I99) of the ICD-10. In this Chapter is included the following subgroup of diseases: ischaemic heart diseases (I20-I25), cerebrovascular diseases (I60-I69) and hypertensive diseases (I10-I15). A premature death was defined as a death occurring among people aged 30–69 years old, considering the life expectancy in the countries of the Americas [6] and the WHO proposal on NCDs global targets and indicators [4]. The criterion to include countries in this study was to have time-series available for the study period, 2000–2010 (unless otherwise specified). A total of 43 countries were included.

### Data analysis

The focus of this analysis is the population-based trend-series. A total of 6,133,666 deaths were analyzed. First, a descriptive analysis was carried out to characterize the current status of the CVD premature mortality in each country and in the Americas, using the information for the latest year available. The number of deaths, proportions and age-adjusted rates are presented for all countries included in this study.

A subsequent trend analysis was undertaken using the Join Point Regression Program, version 4.04 (May 2013), from the National Cancer Institute [12]. The dependent variable was the age-adjusted rate with year as the independent variable. The analysis focused on providing estimates of the average annual percentage of change (AAPC) and confidence intervals. The AAPC is considered significant when the slope is different from "zero" at alpha = 0.05. The tests of significance use a Monte Carlo Permutation method. The number of randomly permuted data sets for permutation tests was set at 4,499, in order to obtain greater consistency in the p-values. For the trend analysis, the following countries in the Caribbean with small population and number of deaths were grouped as “Caribbean Island” to avoid misinterpretation due to fluctuation of rates: Anguilla, Antigua & Barbuda, Aruba, Bahamas, Barbados, Belize, Bermuda, Cayman Islands, Dominica, French Guiana, Grenada, Guadeloupe, Martinique, Montserrat, Saint Kitts & Nevis, Saint Lucia, Saint Vincent and the Grenadines, Turks & Caicos Islands, Virgin Islands (United Kingdom) and Virgin Islands (United States). In addition these countries are close geographically and share similarities regarding economic, social and cultural aspects.

The quality of information of each country was assessed by verifying the integrity and consistency of data in addition to the validation of selected variables (sex, age and underlying cause of death). The proportion of under-registered and ill-defined deaths over time was also used as indicators of data quality. As a procedure to ensure comparability of mortality statistics among countries an algorithm to correct for under-registration and ill-defined causes was applied if a country had an estimated proportion of under-registration of more than 10%, or a proportion of ill-defined causes of more than 10%, or both, according to a published methodology [13]. The under-registration of deaths estimated for the Region of the Americas around 2010 was 7.6% and ill-defined causes was 3.5%, respectively. These proportions for each country can be found as S1 Table and also on-line [6]. In our study data for 10 countries were corrected.

### Ethical considerations

Since this study analyzed anonymous secondary data on mortality, no ethics approval was required.

## Results

### Current situation of premature mortality due to CVD

Premature mortality due to CVD (ICD10 I00-I99) in the Americas fell by 21% in the period 2000–2010, from 169.0 to 132.7 per 100,000 (20% among men and 23% among women). Mortality from ischemic diseases (ICD10 I20-I25), which represented 44% of all deaths due to CVD in 2010, declined by 25% - 24% among men and 26% among women. In turn, cerebrovascular diseases (ICD10 I60-I69), which accounted for 21% of all CVD deaths in 2010, declined by 27% -26% among men and 28% among women.

Table 1 summarizes the current situation in regard to premature mortality. Guyana, Trinidad and Tobago, the Dominican Republic, Bahamas, and Brazil had CVD premature mortality rates over 200 per 100,000 population, while the average for the Region was 132.7. Furthermore, Canada and Martinique had premature mortality rates up to two times lower than the regional average. Premature mortality from ischemic heart diseases was significantly elevated in Guyana, Trinidad and Tobago, the Dominican Republic, and Venezuela with rates above 100 deaths per 100,000 population. Meanwhile, 12 countries reported rates up to 1.5 times lower than the regional average, viz, Ecuador, Peru, Chile, Uruguay, Argentina, Canada, and several countries of the English-speaking Caribbean. Premature mortality from cerebrovascular diseases was between two and four times higher than the regional average (28.1 per 100,000) in Guyana, Suriname, the Dominican Republic, Paraguay, and Brazil; and it was between two and three times lower in six countries, including Canada and the United States.

**Table 1:** Premature (30–69 years) age-standardized mortality rates from cardiovascular diseases, cerebrovascular diseases and ischaemic diseases in 2010, by sex and country.

Countries	Total population (thousands), 2013	World Bank income group‡	Cardiovascular diseases (I00-I99)			Ischaemic heart diseases (I20-25)			Cerebrovascular diseases (I60-69)		
			Total	Male	Female	Total	Male	Female	Total	Male	Female
**Region of the Americas**	**971,907**	**n/a**	**132.7**	**174.8**	**94.2**	**58.9**	**85.4**	**34.6**	**28.1**	**32.5**	**24.0**
Anguilla	16	n/a	110.6	137.0	93.6	30.5	26.8	36.8	16.2	31.5	0.0
Antigua and Barbuda (*)	90	1	174	232.7	124.9	61	101.4	28.1	30	40.6	21.5
Argentina	41,446	2	140	211.6	80.0	38.2	64.5	16.5	32.2	44.2	21.7
Aruba	109	1	111.4	163.1	69.4	29.9	56.6	8.2	26.4	27.2	26.3
Bahamas	377	1	209.2	277.8	148.5	55.3	84.5	29.4	40.3	46.0	35.6
Barbados	289	1	126.4	181.0	79.8	25.2	37.9	14.5	30.4	44.5	18.6
Belize	332	2	172.5	212.0	134.5	79.6	91.9	67.8	36.5	52.9	20.8
Bermuda	69	1	60	87.4	32.3	16.3	33.7	0.0	8.5	4.5	12.1
Brazil	200,362	2	199.9	252.4	153.4	73.2	101.3	47.9	55.7	65.7	47.1
Canada (*)	35,182	1	65	94.2	36.1	40	63.0	17.6	9	10.4	7.9
Cayman Islands	54	1	38.4	35.0	42.6	10.6	22.0	0.0	6.7	6.7	6.7
Chile	17,620	1	91.6	129.7	57.8	34.6	55.7	15.9	28.4	35.8	21.9
Colombia	48,321	2	146.9	187.2	111.3	76.4	107.5	49.1	34.6	36.5	32.7
Costa Rica	4,872	2	98.8	130.2	67.5	53.5	76.8	31.1	15.4	17.8	12.4
Cuba	11,266	2	154.1	195.0	114.3	72.7	99.5	46.6	38.3	43.4	33.5
Dominica	73	2	120.6	138.8	100.7	28	35.4	19.9	30	36.2	24.1
Dominican Republic	10,404	2	238.5	285.3	191.5	117	148.5	85.3	69.2	77.8	61.1
Ecuador	15,738	2	108.9	134.9	82.9	17.9	26.4	10.1	29	31.4	26.4
El Salvador	6,340	3	118.4	148.3	94.4	50.4	67.8	36.9	19.8	24.3	15.9
French Guiana (*)	249	n/a	77	107.9	43.2	12	19.0	3.2	19	27.2	10.3
Grenada	110	2	196.2	264.6	128.4	60.7	85.1	35.7	49.5	56.5	43.7
Guadeloupe (*)	466	n/a	90	135.4	50.9	21	36.9	6.7	24	36.5	13.6
Guatemala	15,468	3	95.6	106.9	87.5	40.9	51.4	32.7	26.1	27.3	25.6
Guyana	800	3	437.6	553.4	333.4	157.4	224.4	97.2	128.8	141.6	117.5
Martinique (*)	404	n/a	63	105.3	27.5	12	22.8	2.4	19	34.0	7.0
Mexico	122,332	2	104.1	129.5	80.1	55	76.9	34.4	23.2	25.5	21.0
Montserrat	5	n/a	151	272.8	0.0	151	272.8	0.0			
Nicaragua	6,080	3	129.4	152.6	105.2	55.2	69.3	42.1	36.6	38.3	34.1
Panama	3,864	2	108.5	137.3	78.2	49.7	69.3	29.4	25.6	31.5	20.1
Paraguay	6,802	3	185.5	215.3	151.7	73.9	94.5	50.8	58.7	60.8	57.3
Peru	30,376	2	87.3	111.4	65.4	23.2	34.3	13.4	26.9	32.3	21.7
Puerto Rico	3,688	1	95.2	133.5	62.9	46.5	68.3	28.2	16.4	21.3	12.3
Saint Kitts and Nevis	51	1	155.4	147.8	163.1	45.2	30.4	60.4	32.3	25.6	37.5
Saint Lucia	163	2	189	252.4	130.4	44.5	76.1	17.0	42.6	50.6	36.0
Saint Vincent and the Grenadines	103	2	167.6	184.4	145.2	51.6	74.1	29.0	46.7	49.7	38.5
Suriname (*)	539	2	184	220.9	150.4	65	97.4	35.0	78	81.2	75.8
Trinidad and Tobago (†)	1,341	1	239	336.8	157.8	124	185.9	73.0	49	68.0	33.7
Turks and Caicos Islands (*)	48	1	67.3	84.9	47.9	7.6	14.1	0.0	39.8	47.8	31.3
United States	320,051	1	111.4	153.4	72.1	57.4	85.8	30.8	14.8	17.1	12.7
Uruguay	3,407	1	114	166.8	69.2	36.9	59.9	17.4	31.6	40.8	24.0
Venezuela (*)	30,405	n/a	181.2	238.1	125.4	106.0	148.3	63.9	41.3	48.7	34.6
Virgin Islands (U.S.)	105	1	124.3	168.4	84.1	64.9	97.5	35.1	10.6	10.9	10.4
Virgin Islands (U.K.) (*)	32	2	73.9	101.9	46.1	36.0	55.7	16.1	12.5	14.3	10.9

Note

(*): 2009 mortality data

(†): 2008 mortality data

(‡): Income groups as classified by the World Bank for the 2015 fiscal year (1) high income, (2) upper middle income, (3) lower middle income; n/a: not available.

Sources: Mortality data: PAHO Mortality information system. Pan American Health Organization. 2014; Total population: countries under 300,000 pop: US Census Bureau, otherwise: UN Population Division estimates; Income groups: The World Bank. Country and Lending groups [Online]. 2014. Available at: http://bit.ly/1lgFrYy. Accessed September 22, 2014.

Guyana, Trinidad and Tobago, and the Dominican Republic had mortality from ischemic heart diseases and cerebrovascular diseases in both sexes at least twice as high as the average for the Region. In Brazil, premature mortality from cerebrovascular diseases among both men and women was twice as high as the regional average. Canada has achieved the low rates of premature mortality from ischemic and cerebrovascular diseases among both men and women. (Table 1)

### Trends in premature mortality due to CVD (2000–2010)

Table 2 summarizes the results of the trend analysis for premature mortality due to CVD (ICD10 I00-I99) in the Americas. The region saw a statistically significant reduction of mortality during the decade of study, with a -2.5% average annual rate of change in the last 5 years. Four countries had a significant reduction in mortality of more than -4% annually (Trinidad and Tobago, Suriname, the United States, and Nicaragua). Annual reductions greater than the regional average of -2.5% per year were also observed in Canada, Argentina, Brazil, Ecuador, Puerto Rico, and Uruguay. Significant annual reductions of more than -1.6% per year would be the minimum required to reach the target of a 25% relative mortality reduction by 2025, if this indicator consisted exclusively of the cardiovascular component. This rate was observed in Cuba, Colombia, Chile, and Venezuela.

**Table 2:** Cardiovascular disease premature age-standardized mortality rate trends, by sex and country.

Countries	Time-series	Annual Average Percentage Change (AAPC) for the last 5 years of the time series								
		Total			Males			Females		
		AAPC	UCI	LCI	AAPC	UCI	LCI	AAPC	UCI	LCI
**Region of the Americas**	**2000–2010**	**-2.5**	**-2.7**	**-2.2**	**-2.3**	**-2.6**	**-2.1**	**-2.7**	**-3**	**-2.4**
Argentina	2000–2011	-2.9	-3.5	-2.4	-2.6	-3.1	-2.1	-3.2	-3.9	-2.5
Brazil	2000–2011	-2.8	-3.3	-2.3	-2.6	-3.1	-2.1	-3	-3.5	-2.5
Canada	2000–2009	-3.7	-4.1	-3.2	-3.6	-4.1	-3.1	-4	-4.5	-3.4
**Caribbean Islands (** * **)**	**2000–2009**	**-2.3**	**-5.5**	**1.0**	**-1.9**	**-5**	**1.4**	**-3.1**	**-6.9**	**0.9**
Chile	2000–2011	-1.6	-2.0	-1.2	-1.2	-1.5	-0.8	-2.7	-3.2	-2.1
Colombia	2000–2010	-1.9	-2.4	-1.5	-1.2	-1.6	-0.7	-3	-3.6	-2.5
Costa Rica	2000–2011	-1.0	-1.8	-0.1	-0.6	-1.9	0.8	-2.8	-3.9	-1.8
Cuba	2001–2011	-2.2	-2.7	-1.6	-1.8	-2.5	-1.2	-2.7	-3.3	-2.1
Dominican Republic	2000–07, 2009–10	-0.4	-1.1	0.3	-0.1	-0.8	0.5	-0.7	-1.5	0.2
Ecuador	2000–2011	-2.8	-3.7	-1.9	-1.7	-2.4	-0.9	-3.8	-4.4	-3.2
El Salvador	2000–2011	-0.2	-1.9	1.5	0.9	0	1.8	-2.7	-3.6	-1.8
Guyana	2001–2010	1.3	-0.2	2.8	0.4	-1.9	2.8	0.2	-1.1	1.4
Mexico	2000–11	-0.9	-3.0	1.3	-0.2	-0.6	0.2	-1.7	-3.3	-0.1
Nicaragua	2000–2011	-4.4	-5.3	-3.5	-4.1	-5.1	-3.2	-5	-6.1	-3.9
Panama	2000–2011	-0.3	-0.9	0.3	0	-0.7	0.7	-0.8	-1.5	0
Paraguay	2000–2011	-3.1	-6.6	0.7	-0.8	-1.6	-0.1	-1.8	-2.5	-1
Peru	2000–2010	-2.1	-3.1	-1.1	-1.7	-2.9	-0.4	-2.7	-3.6	-1.8
Puerto Rico	2000–2010	-2.7	-3.5	-1.8	-2.4	-3.6	-1.2	-3.1	-3.8	-2.5
Suriname	2000–2008	-4.6	-6.8	-2.4	-4.9	-10.1	0.7	-5.5	-6.8	-4.1
Trinidad and Tobago	2000–2008	-4.8	-6.0	-3.7	-3.9	-5.4	-2.4	-6.3	-7.4	-5.2
United States	2000–2010	-4.4	-4.9	-4.0	-2.8	-2.9	-2.6	-3.1	-3.4	-2.9
Uruguay	2000–2010	-2.9	-3.0	-2.7	-3.1	-3.7	-2.5	-3.9	-5.2	-2.6
Venezuela	2000–2009	-1.8	-2.2	-1.3	-0.2	-1.4	1	-2.5	-3.2	-1.8

Notes

AAPC: Annual average percentage change

UCI: Upper confidence interval

LCI: Lower confidence interval

*: Caribbean Islands include Anguilla, Antigua and Barbuda, Aruba, Bahamas, Barbados, Belize, Bermuda, Cayman Islands, Dominica, French Guiana, Grenada, Guadeloupe, Martinique, Montserrat, Saint Kitts and Nevis, Saint Lucia, Saint Vincent and the Grenadines, Turks and Caicos Islands, Virgin Islands (United Kingdom), and Virgin Islands (United States).

Source: PAHO Mortality information system. Pan American Health Organization. 2014.

Premature mortality from CVD among men showed a statistically significant reduction in the last 5 years, at a rate of more than -3% per year in three countries with very different mortality profiles: Trinidad and Tobago (which still has one of highest premature mortality rates in the Region); Canada (which has one of the lowest rates in the Americas); and Uruguay (which has rates close to the regional average). The United States, with a premature mortality rate in the mid-range, and Argentina and Brazil, which have relatively high rates have for the regional context, all achieved annual reductions greater than 2.5%. Also noteworthy are the countries that did not manage to significantly reduce premature mortality among men during that decade, notably Mexico, Costa Rica, Venezuela, Dominican Republic, Panama, Guyana, and El Salvador.

As regards premature mortality due to CVD in women, a sizeable group of countries achieved statistically significant average annual reductions above -2.5%. Trinidad and Tobago (-6.3%) and Suriname (-5.5%) recorded the greatest decline, although both countries had very high rates at the beginning of 2000 (≥ 200 per 100,000 population). Canada and Uruguay are again among the best performing countries, with reductions of approximately -4% per year. Ecuador, Argentina, United States, and Puerto Rico reduced premature mortality from CVD at a rate higher than -3% per year. However, Guyana, the Dominican Republic, and Panama did not achieve the required 1.6% annual reduction, and in other countries, such as Mexico and Paraguay, these reductions were very slight.

Furthermore, one fact that stands out in this analysis is the gap between men and women. The Region saw premature mortality from CVD among women drop at an average annual rate of 2.7% while only 2.3% among men. In this regard, the varied performance of male and female mortality in countries such as El Salvador, Venezuela, and Costa Rica is noteworthy. In these countries, women had statistically significant reductions in mortality ranging between 2.5% and 2.8% annually in the last five years, whereas the rates remained stable for men. In Colombia and Chile, both men and women reduced mortality in the last 5 years, while for women the rate declined almost three times than among men.

## Discussion

Beyond its history and traditions, the Region of the Americas is extraordinarily diverse; therefore any examination of mortality trends must take account of this historical and social heterogeneity. The reduction of premature mortality due to CVD observed during the 2000–2010 decade coincided with a period of economic growth in a majority of the countries, accompanied by a moderate reduction in poverty and greater emphasis on social services [14]. Despite this progress, PAHO recognizes there is still unequal access to services and poor or insufficient distribution of public health expenditures, with high out-of-pocket costs [8].

In order to reduce premature mortality due to CVD by 25% before 2025 mortality must drop by an average of around 1.6% per year between 2010 and 2025. Given the size of the population, the Region’s chances of reaching this goal depend on the performance of the three countries with populations greater than 100 million, which together account for 66% of the Region’s population (the United States, Brazil, and Mexico), and the countries with populations greater than 30 million (Colombia, Argentina, Canada, and Peru). All together, these seven countries account for 80% of the population of the Region.

The United States will meet the 25% reduction target before 2025 if the current trend in premature mortality due to CVD continues. In order to achieve this, a plan has been proposed for an additional reduction of cardiovascular mortality of 20% during the 2010–2020 time period [15], including implementation of the “Million Hearts” initiative [16], which focuses on the very pragmatic “ABCS”: Aspirin therapy, Blood pressure control, Cholesterol control, and support for Smoking cessation. The great handicap this country faces is that, as has been reported [17], obesity and diabetes trends observed in recent years can impact negatively on mortality due to CVD.

In the last decade Brazil has made enormous investments in health [18] and has reduced premature cardiovascular mortality. In order to ensure that mortality continues to decline, more effective control of hypertension is needed, and more attention must be paid to social class and regional disparities in cardiovascular mortality. Mortality rates from cerebrovascular diseases remain high [19], which may be one of the country’s greatest challenges.

Mexico presents a unique situation in terms of mortality caused by CVD. Despite the fact that premature mortality due to CVD is below the regional average, it did not decline further among men or women in the last 10 years. This is further complicated by the fact that premature mortality attributed to diabetes is very high (ICD10 E10-E14) [20]. It is likely that in the case of Mexico coding algorithms for cause of death may give preference to diabetes. Although new studies must be done to understand this dynamic, it is clear that it requires urgent responses, especially in the area of public health to contain the obesity epidemic [21], which most certainly underlies the diabetes problem and all the vascular illness in that country. Also needed are actions to reduce inequities and optimize the performance of the health services, which are central issues of health system reform [22].

Canada began to reduce CVD mortality in the late 1960s. By the early 2000s it already had low mortality, yet premature CVD mortality continued to decline faster than in any other country of the region. Success in managing and controlling hypertension was a key part of this achievement [23]. Additional efforts to control hypertension will be needed if CVD mortality rates are to keep going down.

Although trends in the most populous countries of the Region imply that it will be possible to reach the target of a 25% reduction in relative mortality by 2025, we must also consider the CVD trends in the rest of the Region. In Central America, where some of the poorest countries of the Region are found, there is a dual burden of disease (both communicable and non-communicable). The health infrastructure is under-developed in several of these countries, access to services is difficult [8], and mortality rates are slightly below the regional average. Generally, this sub-region has not been achieving substantial reductions in premature mortality.

The countries of the English-speaking Caribbean heterogeneous and the small size of their populations makes it difficult to obtain precise estimates. The most serious case is Guyana, which has a high premature mortality rate which is rising further. Premature mortality in Trinidad and Tobago, on the other hand, has been declining rapidly, but it is still high. It remains to be seen how the increasing prevalence of obesity and diabetes will affect premature mortality caused by CVD in Central America and Caribbean. In addition, data from countries with small populations, and therefore very few deaths, should be viewed with caution.

There is scant information available to determine to what degree changes in the population’s cardiovascular risk profile, or progress made in the clinical management of CVDs, is responsible for the reduction in premature mortality seen in most of the countries of the Region. However, when the data was reviewed in the U.S.A, approximately half of the reduction in mortality from heart disease between 1980 and 2000 was attributed to reductions in the main risk factors, while the other half was attributed to evidence-based medical therapies [24]. In Latin America and the Caribbean, the most recent profiles on risk factors for CVD are quite heterogeneous [25], while there has been a considerable increase in rates of obesity and diabetes [26–28]. This phenomenon could eclipse progress the Region has made in the fight against tobacco [29] and the moderate improvements in access to health services [8].

Nonetheless, the clinical and public health priorities for continued reduction of CVD mortality in this Region have been agreed upon by the main regional stakeholders [30]. Thus, there is still a need to step up tobacco control actions, since the estimated adult smoking rate is as high as 22%, in a Region where 30 of 35 countries have ratified the WHO Framework Convention on Tobacco Control [29]. Actions aimed at reducing sodium consumption have recently begun. The average daily intake of this mineral in selected countries of the Region is between 9–12 mg, double the recommended amount [31]. Hypertension affects 20–40% of the adult population, and its control rate (total hypertensive with blood pressure under 140/90) is still unacceptably low [32–34]. Lastly, although multiple-drug therapy is one of the most cost-effective ways to reduce mortality from CVD [5], many countries still do not include this intervention in the benefits offered through primary care services [35].

Some limitations have to be taken into consideration to interpret these findings. First, although the data was corrected for under-registration of deaths and ill-defined causes, the rates from some countries, especially the less resourced ones, can still be affected by the problem. The second is related to the analysis by sub-groups of causes because the classification of the underlying cause of death can vary among countries. Some countries have a high proportion of deaths classified as heart failure and others have a high proportion of deaths classified as hypertensive diseases which could potentially be cerebrovascular diseases. Finally, the comparison of current rates from less populous countries should be done carefully due to the expected fluctuation of rates. On the other hand, one of the strengths of this study is to show trend data on premature mortality to allow countries to take action to achieve the global reduction target.

In summary, trends in premature mortality due to CVD observed in last decade in the Americas, together with the commitments made in recent years [36], would indicate that if these trends continue, the Region as a whole and a majority of its countries will be able to reach the goal of a 25% relative reduction in premature mortality even before 2025. This could well be considered a predictable and unsurprising scenario, particularly for a Region such as the Americas which has made a commitment to move toward universal health coverage after a decade of many health’s achievements [37]. This means, hopefully, that scientific progress in the prevention of CVD and in clinical medicine will be able to reach those who need them most. Finally, the question naturally arises, should we be satisfied with a 25% reduction by 2025, or should we aim for more?

## Supporting Information

S1 Fig Cardiovascular diseases premature age-standardized mortality rates, both sexes, by country. (DOCX)

S1 Table Percentage of ill-defined and unknown causes of death and mortality under-registration (2011), by country and subregion. (DOCX)
