# Supplementary material for: Premature Mortality from Cardiovascular Disease in the Americas – Will the Goal of a Decline of “25% by 2025” be Met?
Source: PLoS One. 2015 Oct 29;10(10):e0141685. doi: 10.1371/journal.pone.0141685 (PMC4626103; doi:10.1371/journal.pone.0141685)
Supplement: S1 Fig — (DOCX) [file pone.0141685.s001.docx]

**S1 Figure. Cardiovascular diseases premature age-standardized mortality rates, both sexes, by country.**
