# Supplementary material for: Premature Mortality from Cardiovascular Disease in the Americas – Will the Goal of a Decline of “25% by 2025” be Met?
Source: PLoS One. 2015 Oct 29;10(10):e0141685. doi: 10.1371/journal.pone.0141685 (PMC4626103; doi:10.1371/journal.pone.0141685)
Supplement: S1 Table — (DOCX) [file pone.0141685.s002.docx]

**S1 Table. Percentage of ill-defined and unknown causes of death and mortality under-registration (2011), by country and subregion.**

| Subregion/country | Ill-defined and unknown causes of death (%), 2011 | Mortality under-registration (%), 2011 |
| --- | --- | --- |
| **The Americas** | **3.5** | **7.6** |
| **North America** | **1.5** | **3.4** |
| Bermuda | 2.4 | 9.3 |
| Canada | 0.9 | 4.0 |
| United States of America | 1.6 | 3.3 |
| **Latin America & the Caribbean** | **4.7** | **10.1** |
| **Latin America** | **4.8** | **10.1** |
| Mexico | 1.8 | - |
| **Central American Isthmus** | **6.6** | **15.9** |
| Belice | 1.1 | - |
| Costa Rica | 1.2 | 9.3 |
| El Salvador | 16.3 | 23.1 |
| Guatemala | 7.3 | 10.4 |
| Honduras | … | … |
| Nicaragua | 1.7 | 32.0 |
| Panama | 3.1 | 11.0 |
| **Latin Caribbean** | **2.6** | **21.2** |
| Cuba | 0.8 | - |
| Dominican Republic | 4.3 | 52.0 |
| French Guiana | 9.4 | 14.1 |
| Guadeloupe | 9.4 | 3.8 |
| Haiti | … | … |
| Martinique | 11.3 | 9.2 |
| Puerto Rico | 1.0 | 2.7 |
| **Andean Area** | **2.8** | **21.7** |
| Bolivia (Plurinational State of) | … | … |
| Colombia | 2.1 | 20.3 |
| Ecuador | 9.4 | 16.7 |
| Peru | … | 36.7 |
| Venezuela (Boliviarian Republic of) | 0.6 | 11.7 |
| Brazil | 6.7 | 8.2 |
| **Southern Cone** | **7.1** | **5.1** |
| Argentina | 8.4 | 1.4 |
| Chile | 2.5 | 5.6 |
| Paraguay | 10.4 | 28.7 |
| Uruguay | 8.4 | - |
| **Non-Latin Caribbean** | **2.9** | **10.8** |
| Anguilla | 1.6 | 9.0 |
| Antigua & Barbuda | 1.1 | 26.9 |
| Aruba | 4.6 | 24.3 |
| Bahamas | 1.9 | 4.7 |
| Barbados | 2.2 | 5.5 |
| Cayman Islands | 4.0 | 39.8 |
| Curaçao | … | … |
| Dominica | 1.9 | 0.2 |
| Grenada | 1.7 | 6.7 |
| Guyana | 0.8 | 1.6 |
| Jamaica | … | … |
| Montserrat | - | - |
| Saint Kitts & Nevis | 1.1 | - |
| Saint Lucia | 4.6 | 14.8 |
| Saint Vincent & the Grenadines | 4.3 | - |
| Sint Maarten (Dutch part) | … | … |
| Suriname | 8.3 | 25.5 |
| Trinidad & Tobago | 1.9 | 11.0 |
| Turks & Caicos Islands | 8.2 | 26.9 |
| Virgin Islands (UK) | 10.0 | 19.3 |
| Virgin Islands (US) | 3.6 | 7.9 |

Note: -: Magnitude zero ;…: Information not available

Source: Pan American Health Organization/World Health Organization. Communicable Diseases and Health Analysis: Health Situation in the Americas: Basic Indicators 2014. Washington, D.C., USA, 2014. Accessed March 1, 2015. Available at: <http://www.paho.org/hq/index.php?option=com_docman&task=doc_view&gid=23080&Itemid=270&lang=en>.
